# Is triple artemisinin-based combination therapy necessary for uncomplicated malaria?

**DOI:** 10.1016/S1473-3099(22)00283-3

**Published:** 2022-06-01

**Authors:** Rob W van der Pluijm, Thomas J Peto, Mainga Hamaluba, James J Callery, Rupam Tripura, Nicholas J White, Arjen M Dondorp

**Affiliations:** Mahidol-Oxford Tropical Medicine Research Unit, Faculty of Tropical Medicine, Mahidol University, Bangkok 10400, Thailand; KEMRI Wellcome Trust Research Programme, Kilifi, Kenya; Mahidol-Oxford Tropical Medicine Research Unit, Faculty of Tropical Medicine, Mahidol University, Bangkok 10400, Thailand

We thank Chengchao Xu and colleagues^1^ and Charlotte Rasmussen and Pascal Ringwald^2^ for their interest in our studies^3,4^ on triple antimalarial combination therapies (TACTs). TACTs are developed to counter the increasing problem of *Plasmodium falciparum* resistance to artemisinins and their partner drugs in artemisinin combination therapies (ACTs).

Xu and colleagues suggest that rotating ACTs with different partner drugs, adjusting the time course of artemisinin treatments, or exploring improved artemisinin derivatives would be better strategies to counter these resistance problems. Drug rotation is what has been happening already, albeit reactively, but it is operationally challenging. Experience from several countries in southeast Asia suggests that changing first-line antimalarial therapy often takes several years to implement, even when treatment failure rates have risen. Meanwhile, artemisinin resistance facilitates the emergence and selection of partner-drug resistance, jeopardising the small number of available ACT partner drugs. Combining the potent, but short-acting, artemisinin component with two slower, but longer-acting, matching partner drugs in TACTs provides mutual protection against resistance.^5^ The alternative of prolonging the standard 3-day ACT course might improve treatment efficacy but for several ACTs this would require a shift to a second ACT halfway through the treatment course to avoid partner-drug accumulation and toxicity. This more complex treatment regimen would likely compromise treatment adherence. Unfortunately, improved artemisinin derivatives and other new antimalarial compounds are not expected within the next 5 years.

We agree that reducing adverse effects and increasing cost-effectiveness are essential in the development of TACTs. The expected longer therapeutic lifespan of TACTs compared with ACTs will also be a crucial element of this cost−benefit analysis.

Rasmussen and Ringwald state that well matched (triple) combinations might be the future of malaria treatment. Delaying antimalarial drug resistance with TACTs has become an increasingly relevant consideration with the emergence of artemisinin resistance in Africa.^6^ Ideally, a triple combination would include only drugs that are individually curative, and without existing resistance. However, the current reality is a choice between a small number of available antimalarials. Artemether−lumefantrine−amodiaquine was studied because of the well matched pharmacokinetic profiles of the partner drugs and the in-vitro counteracting resistance mechanisms.^7^ In addition, the combination has shown excellent safety and efficacy in areas of highly resistant falciparum malaria in the Greater Mekong subregion, in which the number of cases is falling but elimination has not yet been achieved.^4,5^ Artemether−lumefantrine−amodiaquine is now being further evaluated in a large randomised trial in Africa and a fixed-dose combination is in development.
